# The expression level of miR-18b in hepatocellular carcinoma is associated with the grade of malignancy and prognosis

**DOI:** 10.1186/1471-2407-13-99

**Published:** 2013-03-04

**Authors:** Yoshiki Murakami, Akihiro Tamori, Saori Itami, Toshihito Tanahashi, Hidenori Toyoda, Masami Tanaka, Weihong Wu, Nariso Brojigin, Yuji Kaneoka, Atsuyuki Maeda, Takashi Kumada, Norifumi Kawada, Shoji Kubo, Masahiko Kuroda

**Affiliations:** 1 Department of Hepatology, Graduate School of Medicine Osaka City University, Osaka, 545-8585, Japan; 2 Division of Gastroenterology, Department of Internal Medicine, Kobe University Graduate School of Medicine, Kobe, 650-0017, Japan; 3 Department of Gastroenterology, Ogaki Municipal Hospital, Ogaki, 503-8502, Japan; 4 Department of Molecular Pathology, Tokyo Medical University, Tokyo, 160-8402, Japan; 5 Department of Surgery, Ogaki Municipal Hospital, Ogaki, 503-8502, Japan; 6 Department of Hepato-Biliary-Pancreatic Surgery, Graduate School of Medicine, Osaka City University, Osaka, 545-8585, Japan; 7 Present address: Laboratory of Genome Technology, Human Genome Center, Institute of Medical Science, The University of Tokyo, Tokyo, 108-8679, Japan

**Keywords:** Hepatocellular carcinoma, Histological differentiation, miRNA, Biomarker, TNRC6B

## Abstract

**Background:**

Many studies support the hypothesis that specific microRNA (miRNA) expression in various human cancers including hepatocarcinogenesis is closely associated with diagnosis and prognosis. In hepatocellular carcinoma (HCC), malignancy level is related to the degree of histological differentiation.

**Methods:**

In order to establish a novel biomarker that can determine the degree of malignancy and forecast patient prognosis, we performed a microarray analysis to investigate the miRNA expression profiles in 110 HCC which were comprised of 60 moderately, 30 poorly, and 20 well differentiated HCC.

**Results:**

We found that the expression of 12 miRNAs varied significantly according to the degree of histological differentiation. Particularly, miR-18b expression in poorly differentiated HCC was significantly higher than in well differentiated HCC. Based on miRanda and Targetscan target search algorithms and Argonaute 2 immunoprecipitation study, we noted that miR-18b can control the expression of trinucleotide repeat containing 6B (TNRC6B) as a target gene. Additionally, in two hepatoma cell lines, we found that over-expression of miR-18b or down-regulation of TNRC6B accelerated cell proliferation and loss of cell adhesion ability. Finally, we observed that after surgical resection, HCC patients with high miR-18b expression had a significantly shorter relapse-free period than those with low expression.

**Conclusions:**

miR-18b expression is an important marker of cell proliferation and cell adhesion, and is predictive of clinical outcome. From a clinical point of view, our study emphasizes miR-18b as a diagnostic and prognostic marker for HCC progression.

## Background

Hepatocellular carcinoma (HCC) is the third most common cause of death from cancer worldwide [1]. The most frequent etiologies of HCC are chronic hepatitis B and C (CHB, CHC), and alcoholic liver disease [2]. Although recent advances in functional genomics provide a deeper understanding of hepatocarcinogenesis [3,4], the molecular pathogenesis of HCC remains rather unclear. Indeed, the clinical heterogeneity of HCC and the lack of good diagnostic markers and treatment strategies have rendered this disease a major challenge.

Cell differentiation and drug-induced differentiation of tumor cells into benign or normal cells, are important targets for anticancer chemotherapy [5]. Cellular differentiation in HCC progresses from non-tumor tissue to well-differentiated cancerous tissue [6]. As such, along with other clinical factors, the degree of histological differentiation in HCC is closely related to clinical course. Tumor-free survival rates have shown that moderately or poorly differentiated HCC is a significant risk factor for recurrence [7].

It is widely known that miRNAs are important in the control of numerous biological processes, such as development, differentiation, proliferation and apoptosis [8]. Altered miRNA expression has been observed in a large variety of HCC and a correlation has been found between miRNA expression and histological differentiation [9,10]. The expression level of miR-26 was associated with hepatocarcinogenesis and response of interferon therapy [11]. Moreover, the hepatic miRNA expression pattern that existed in CHC patients before anti-viral therapy is associated with the outcome of pegylated interferon and rivabirin combination therapy [12]. Additionally, aberrant expression of miRNAs particularly, miR-199a, miR-199a*, miR-200a, and miR-200b has been closely associated with the progression of liver fibrosis in both human and mouse [13]. Recently the expression level of miR-122 was associated with not only hepatocarcinogenesis but liver homeostasis and essential liver metabolism [14,15]. Among others, miR-18 which is intimately associated with the occurrence and progression of different types of cancer have also been implicated [16]. In other research, miRNA expression profile was associated with vascular invasion, the value of alpha-fetoprotein, and large tumor size [17].

Given miRNA’s importance in liver pathology, we sought to evaluate the diagnostic and prognostic significance of miRNA expression in HCC and to determine the functional implication of miRNAs deregulation in the development of liver cancer. As a part of this process, we profiled miRNA expression according to the degree of histological differentiation of HCC, and established a novel biomarker for determining HCC malignancy degree. Using our findings, we will show that miR-18b repression in HCC correlates with clinically relevant parameters such as histological differentiation status, and that the loss of miR-18b expression correlates with distinct gene expression profiles characteristic of tumor progression (that is, suppression of hepatic differentiation phenotype and gain of metastatic properties).

## Methods

### Sample preparation

110 hepatocellular carcinoma tissue samples were obtained by surgical resection (Additional file 1: Table S1). All patients or their guardians provided written informed consent, and Osaka City University, Ogaki Municipal Hospital and Kyoto University Graduate School and Faculty of Medicine’s Ethics Committee approved all aspects of this study in accordance with the Helsinki Declaration.

### RNA preparation and miRNA microarray

Total RNA from cell lines or tissue samples was prepared using a mirVana miRNA extraction Kit (Ambion, Austin, TX, USA) according to the manufacturer’s instruction. To detect miRNA, 100 ng of RNA was labeled and hybridized using the Human microRNA Microarray Kit (Rel 12.0) (Agilent Technologies, CA, USA) according to manufacturer’s protocol for use with Agilent microRNA microarrays Version 1.0. Hybridization signals were detected with Agilent DNA microarray scanner G2505B and the scanned images were analyzed using Agilent feature extraction software (v9.5.3.1). Data were analyzed using GeneSpring GX 7.3.1 software (Agilent Technologies) and normalized as follows: (i) Values below 0.01 were set to 0.01. (ii) Each measurement was divided by the 75th percentile of all measurements from the same species. All data were deposited in NCBI’s Gene Expression Omnibus and are accessible through GEO Series accession number GSE31164.

### Real-time qPCR for human miRNA

To detect miRNA level by real-time qPCR, TaqMan® microRNA assay (Applied Biosystems) was used to quantify the relative expression level of miR-18b (assay ID. 002217); U18 (assay ID. 001204) was used as an internal control. cDNA was synthesized using the Taqman miRNA RT Kit (Applied Biosystems). Total RNA (10 ng/ml) in 5ml of nuclease free water was added to 3 ml of 5× RT primer, 10× 1.5 μl of reverse transcriptase buffer, 0.15 μl of 100 mM dNTP, 0.19 μl of RNase inhibitor, 4.16 μl of nuclease free water, and 50U of reverse transcriptase in a total volume of 15 μl. The reaction was performed in triplicate for 30 min at 16°C, 30 min at 42°C, and 5 min at 85°C. Chromo 4 detector (BIO-RAD) was used to detect miRNA expression.

### Cell lines and miRNA or DNA transfection

The human hepatoma cell lines Huh-7, Li7 and human embryonal kidney cells lines 293FT were obtained from Japanese Collection of Research Bioresources cell bank. Cells were maintained in D-MEM (Invitrogen, Carlsbad, CA, USA) with 10% fetal bovine serum and plated in 6-well plates the day before transfection, then grown to 70% confluence. Cells were transfected with 12.5 pmol/l of Silencer® negative control siRNA (Ambion), siTNRC6B s; 5′-gggacaaggaggaaagaaatt-3′, as; 5′-uuucuuuccuccuuguccctt-3′ (Hokkaido System Science, Sapporo, Japan) or double-stranded mature miR-18b (Hokkaido System Science) using lipofectamine RNAiMAX (Invitrogen). Cells were also transfected with 1 μg/μl of negative control cDNA empty vector or total TNRC6B expression vector (Addgene) using Fugene® (Roche). TNRC6B complete plasmid set was obtained from the non-profit repository AddGene (http://www.addgene.com). Cells were harvested 48 hr after transfection.

### Real-time qPCR

cDNA was synthesized using the Transcriptor High Fiderity cDNA synthesis Kit (Roche, Basel, Switzerland). Total RNA (2 μg) in 10.4 μl of nuclease free water was added to 1 μl of 50 mM random hexamer and denatured for 10 min at 65°C. The denatured RNA mixture was added to 4 μl of 5× reverse transcriptase buffer, 2 μl of 10 mM dNTP, 0.5 μl of 40U/μl RNase inhibitor, and 1.1 μl of reverse transcriptase (FastStart Universal SYBR Green Master Roche) in a total volume of 20 μl. The reaction was run in triplicate for 30 min at 50°C (cDNA synthesis), and five min at 85°C (enzyme denaturation). Chromo 4 detector (BIO-RAD, Hercules, CA, USA) was used to detect mRNA expression. The primer sequences was as follows TNRC6B s; 5′-acaagtgacaggagcgctgctg-3′, as; 5′- ccatgtcagacccgtctacaat-3′, and β-actin s; 5′-ccactggcatcgtgatggac-3′, as; 5′-tcattgccaatggtgatgacct-3′. Assays were performed in triplicate, and the expression levels of target genes were normalized to the expression of the β-actin gene (internal control), as quantified by real-time qPCR.

### Transient transfection and luciferase assay

To generate the TNRC6B 3′-UTR luciferase reporter vectors, the TNRC6B 3′-UTR segments were generated (Additional file 1: Table S3) and inserted into the pMIR-REPORT Luciferase vector between SpeI and HindIII sites (Ambion). Wild and mutant type reporter vectors with miR-18b complementary sites were confirmed by sequencing. Huh7.5 cells (5×10^4^ cell/well) were transfected into 24-well dishes with DMRIE-C (Invitrogen), 10pmol of double stranded mature miR-18b, Antisense oligonucleotide of miR-18b, or negative control of RNA and 0.25 μg wild or mutant type reporter vector. After 48 h, the transfected cells were harvested and lysed, and their luciferase activity was measured with a Dual-Luciferase Reporter Assay System kit (Promega). The experiments were repeated at least three times.

### Co-immunoprecipitation with Ago2

A cell lysate from RNA-induced silencing complex (RISC), was collected using microRNA Isolation Kit, Human Ago2 (Wako, Osaka, Japan) according to the manufacturer’s instruction. Briefly, 48 hr after transfection with 12.5 pmol/L of double stranded mature miRNA, 5×10^6^ cells in 6 cm dish were washed with PBS and harvested by trypsinization. The cell pellet was re-suspended with the gentle pipetting in 1ml of cell lysis buffer (microRNA Isolation Kit). The cell suspension was incubated for 10 minutes on ice, and was then centrifuged at a force of 20000 g for 20 minutes at 4°C. The cells were suspended in PBS and mixed with beads conjugated human Argonaute2 (hAgo2) monoclonal antibody for 2 hr at 4°C. RNA from Ago2-immunoprecipitation fraction was then extracted using mirVana miRNA extraction kit.

### *In situ* hybridization for miR-18b and immunohistochemistry for TNRC6B

Eight paraffin-embedded tissue samples (case 64, 108, 248, 261, 274, 277, 310 and 333) were used (Additional file 1: Table S1). We generated both a locked nucleic acid (LNA) − modified probe for miR-18b (5′- taaggtgcatctagtgcagttag-3′) and a scrambled negative control sequence (5′-gtgtaacacgtctatacgccca-3′: miRCURY-LNA detection probe, Exiqon, Vedbaek, Denmark). *In situ* hybridization utilized a RiboMap *in situ* hybridization kit (Roche Diagnostic) on a Ventana Discovery automated *in situ* hybridization instrument (Roche Diagnostic). For immunohistochemistry of FFPE sections, we used the Ventana HX System Benchmark (Roche Diagnostic). TNRC6B antibody (HPA003180) was purchased from Sigma-Aldrich, St. Louis, MO, USA.

### Estimating positive staining for miR-18b using *in situ* hybridization and immunostaining of TNRC6B

Positive *in situ* hybridization staining and immunostaining were interpreted semi-quantitatively by assessing the intensity and extent of staining on the entire tissue sections observed on the slides, as described previously [18].

### Cell proliferation assay

The cell proliferation assay was performed using XTT® Cell Proliferation Assay Kit (Roche). Briefly, huh7 and Li7 cells (5×105 cells/ml) were spread into 96-well dishes. 12.5 pmol/l of double stranded mature miR-18b, 2′-O-methylated antisense oligonucleotide (ASO) of miR-18b (Hokkaido System Science), siRNA for TNRC6B (Hokkaido System Science) and Silencer® negative control siRNA (Ambion), were transfected with lipofectamine RNAiMAX (Invitrogen). 2 ug of plasmids containing the TNRC6B (Addgene), or empty vector pcDNA3 (Invitrogen) was transfected with FuGENE 6 (Roche). After 24 or 72 hr of transfection, cells were washed twice with PBS, 50ul of XTT labeling mixture was added, and then cells were incubated in a humidified atmosphere for 6 hr at 37°C. After incubation, the absorbance of samples was measured using an ELISA reader at 450–500 nm against a reference wavelength of 650 nm.

### Cell adhesion assay

The cell adhesion assay was carried out using Vybrant® Cell Adhesion Assay Kit (Invitrogen). Briefly, transfection procedure was same as the cell proliferation assays. After 24 or 72 hr of transfection, cells were washed twice with PBS then re-suspended in serum free D-MEM and incubated with 5 μl of the calcein AM stock solution at 37°C for 30 min. Following this, cells (5×10^5^/ml) were washed twice with D-MEM and re-suspended in D-MEM. The calcein-labeled cells were incubated at 37°C. After 120 min, non adherent cells were removed by washing and fluorescence was measured with a fluorescein filter set (absorbance 494 nm, emission 517 nm). We determined the percentage of adhesion by dividing the corrected (background subtracted) fluorescence of adherent cells by the total corrected fluorescence of cells added to each well.

### Statistical analyses

Statistical analyses were performed using Student’s *t*-test; *p* values less than 0.05 were considered statistically significant. Microarray data were also statistically analyzed using ANOVA or Welch’s test and Bonferroni correction for multiple hypotheses testing. Survival analysis was used with Kaplan-Meier survival curve and log-rank tests in the R software environment.

## Results

### Microarray analysis

miRNA expression profiles in 110 HCC were established by microarray analysis (Table 1 and Additional file 1: Table S1) and comprised of 60 moderately, 30 poorly, and 20 well differentiated HCC. We chose miRNAs that were clearly expressed in at least 70% of all samples as determined by numeric analysis. Twelve miRNAs were significantly differentially expressed depending on whether HCC was poorly, moderately or well differentiated according to ANOVA analysis. The expression of miR-221, miR-18a, miR-18b, and miR-423-5p in poorly differentiated HCC were significantly higher than in well differentiated HCC, and 8 miRNAs (miR-455-3p, miR-1914*, miR-100, miR-215, miR-122*, let-7b, miR-22 and miR-99a) in poorly differentiated HCC had significantly lower expression levels than in well differentiated HCC (p < 0.05) (Table 2).

**Table 1:** Clinical background

**histological differentiation**	**number (gender)**	**age (average)**	**viral infection**	**background of HCC**
well	20 (M:15, F:5)	66.5+/−6.3	HBV:2, HCV:18	CH:7, LC:12, NI:1
moderately	60 (M:51, F:9)	66.5+/−8.7	HBV:7, HCV:49, NBNC:3, NI:1	CH:25, LC:30, NI:5
poorly	30 (M:27, F:3)	67.7+/−5.4	HBV:0, HCV:20	CH:14, LC:16

Abbreviation: *NBNC*, neither HBV nor HCV infection, *NI*, no information, *CH*, chronic hepatitis, *LC*, liver cirrhosis.

**Table 2:** Significantly different expression of miRNA according to the histological differentiation

		**histological differentiation**		
miRNA	poor	moderately	well	p-value
hsa-miR-455-3p	0.887	1.000	1.033	0.0012
hsa-miR-221	0.993	1.000	0.800	0.0027
hsa-miR-1914*	0.925	1.000	1.196	0.0025
hsa-miR-18b	1.175	1.000	0.761	0.0064
hsa-miR-100	0.850	1.000	1.000	0.0221
hsa-miR-215	0.954	1.000	1.026	0.0103
hsa-miR-122*	0.905	1.000	1.015	0.0006
hsa-let-7b	0.927	1.000	1.022	0.0178
hsa-miR-22	0.885	1.000	1.010	0.0026
hsa-miR-99a	0.877	1.000	0.976	0.0047
hsa-miR-18a	1.127	1.000	0.893	0.0071
hsa-miR-423-5p	1.087	1.000	0.806	0.0045

The relative expression value of each miRNAs which set moderately differentiation to 1.000 is shown.

### Determining miR-18b target genes

**Figure 1:**
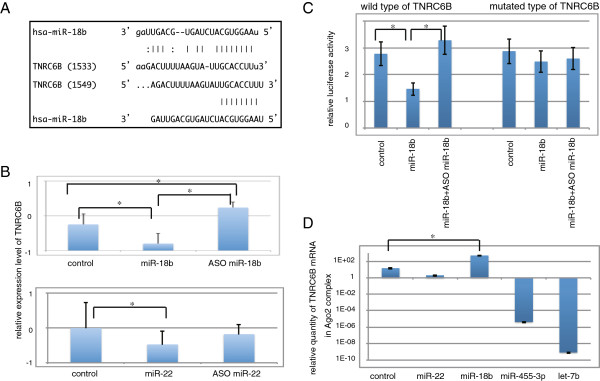
**Process of retrieving target genes of several miRNAs. A**) Homology of the sequence between miRNA and TNRC6B. Complementary of the sequence between miR-18b and TNRC6B gene by miRanda algorithm (upper side) and Targetscan algorithm (lower side). **B**) Changes in TNRC6B expression when miRNA is over-expressed or suppressed are shown means ± SD of three independent experiments. Asterisk indicates a significant difference (p < 0.05). **C**) Transfection of reporter vectors with either the wild (left part) or mutated (right part) TNRC6B 3^′^UTR and miR-18b. The data shown are means ± SD of three independent experiments. Asterisk indicates a significant difference (p < 0.05). **D**) Target confirmation by argonaute 2 (Ago2) immunoprecipitation (IP). When miR-18b exists in RISC, compared with existence of control RNA, TNRC6B is abundantly contained in RISC. TNRC6B RNA was measured by real-time qPCR in 10 ng sample of total RNA from the Ago2-IP fraction. The data shown are means ± SD of three independent experiments. Asterisk indicates a significant difference (p < 0.05).

We then detected the target gene of the 12 miRNAs that were differentially expressed according to the level of HCC differentiation. Homo sapiens trinucleotide repeat containing 6B (TNRC6B) was a common hypothetical target gene in miR-221, miR-18a, miR-18b, miR-423-5p, miR-455-3p, miR-1914*, miR-215, miR-122*, let-7b, and miR-22 using miRanda algorithm. TNRC6B on the other hand, was a common target gene in miR-221, miR-18a, miR-18b, miR-423-5p, and miR-22 using Targetscan. miR-221, miR-18a, miR-18b, miR-423-5p, and miR-22 could recognize TNRC6B as a target gene using both algorithms (Figure 1A)

To clarify the biological links between miRNAs and TNRC6B, we examined the expression pattern of TNRC6B in Huh7 cells by real-time qPCR when expression levels of miR-18a, miR-18b miR-122, miR-221, miR-423-5p, and miR-22 were either over-expressed or suppressed. The result was that low expression of TNRC6B was reflected by over-expression of miR-18b treated with mature miR-18b and vise versa when miR-18b was suppressed with antisense oligonucleotide. The expression pattern of TNRC6B when miR-22 was over-expressed or suppressed was similar to the expression pattern using miR-18b (Figure 1B). However, over-expression of miR-18a, miR-122, and miR-423-5p did not suppress the expression level of TNRC6B and suppression of miR-221 did not induce over-expression of TNRC6B.

Based on our findings, we prepared the reporter gene assay with wild or mutant sequence of the hypothetical binding site of TNRC6B and miR-18b. When miR-18b was co-transfected with wild type of 3′UTR of TNRC6B reporter genes, we observed that luciferase activity was significantly low compared to co-transfecting control RNA or miR-18b plus ASO miR-18b with wild type vector. However, in case of the mutant form of 3’UTR of TNRC6B reporter vector, the luciferase activity was not affected by transfection of any miRNAs (Figure 1C).

Then, we speculated that miR-18b and miR-22 could regulate the expression level of TNRC6B, and to clarify this physiological association, we performed an Ago2-coimmunoprecipitation (Ago2-IP) analysis. Ago2-IP fractionated cell lysates were prepared by transfecting 293FT cells with mature double strand of miR-18b, miR-22, miR-455-3p, let-7b, or a non-specific siRNA which was used as a control RNA. miR-455-3p and let-7b were used as negative controls. The TNRC6B RNA in the Ago2-IP fraction (IP RNA) was quantified by real-time qPCR. The concentration of TNRC6B IP-RNA treated with miR-18b was higher than those treated with the control RNA or double strand of miR-22, miR-455-3p, and let-7b (Figure 1D). Taken together, we concluded that miR-18b can regulate the expression of TNRC6B as a target gene.

### miR-18b and TNRC6B expression correspond to the degree of histological differentiation

**Figure 2:**
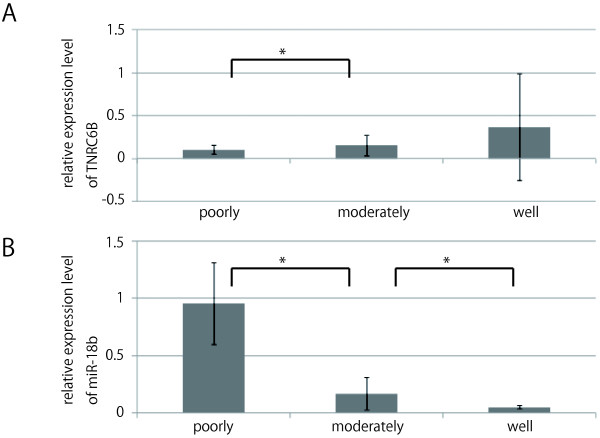
**Expression pattern of TNRC6B and miR-18b according to the histological differentiation.** Expression pattern of TNRC6B and miR-18b according to the degree of histological differentiation by real-time qPCR. Each column represents the relative amount of TNRC6B normalized to the expression level of β-actin or the relative amount of miR-18b normalized to the expression level of U18. The data shown are means ± SD of three independent experiments. Asterisk indicates a significant difference (p < 0.05).

**Figure 3:**
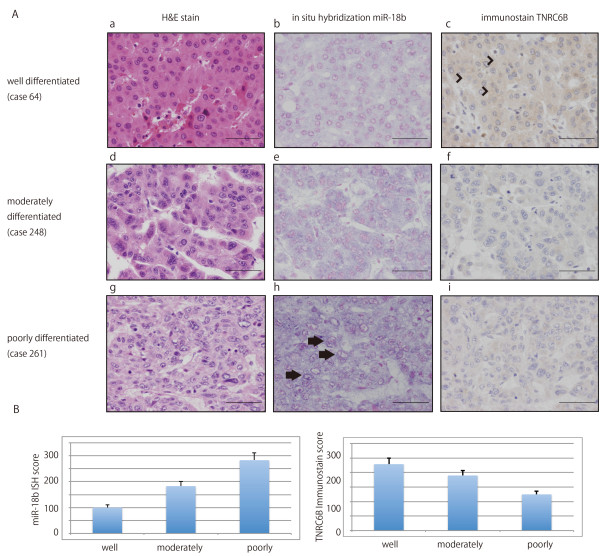
**A) Expression of miR-18b and TNRC6B according to the degree of histological differentiation in HCC.** Well differentiated HCC showed low expression of miR-18b; poorly differentiated HCC showed strong expression of miR-18b. TNRC6B down-regulation in HCC is inversely related to miR-18b expression. HE stain (**a**, **d**, **g**), *in situ* hybridization of miR-18b (**b**, **e**, **h**) and immunohistochemistry of TNRC6B (**c**, **f**, **i**) are shown, respectively. Blue indicates the expression of miR-18b (arrows) and brown indicates the expression of TNRC6B (arrowheads). Bars indicate 100 μm. **B**) Positive cells for miR-18b *in situ* hybridization and TNRC6B immunostain was quantified by each miR-18b *in situ* hybridization and TNRC6B immunostain score system, respectively.

We compared TNRC6B expression in clinical samples with the grade of histological differentiation. TNRC6B expression in poorly differentiated HCC was lower than in well differentiated HCC (Figure 2A). miR-18b confirmed the microarray results using real-time qPCR, while the real-time qPCR result corresponded to the microarray analysis result (Figure 2B). Although the microarray and realtime qPCR results correlated, the expression level of miRNA in both experiments differed. One reason for this difference could be that the base sequences of probe which recognizes miR-18b differ. However, there was no significant correlation between the contra-relation of miR-18b and TNRC6B expression. We then performed *in situ* hybridization using locked nucleic acid (LNA)-modified probes labeled with digoxigenin (DIG) in miR-18b and also immunohistochemistry of TNRC6B in 8 samples (well differentiated: case 64, 108; moderately differentiated: 248, 277, 310, 333; poorly differentiated: case 261, 274). We found high miR-18b and low TNRC6B expression levels in poorly differentiated HCC (case 261), low miR-18b and high TNRC6B expression levels in highly differentiated HCC (case 64), and moderate expression miR-18b and TNRC6B in moderately differentiated HCC (case 248) (Figure 3A, B). The results of the five remaining cases and scrambled LNA study are not shown.

### Aberrant expression of miR-18b and TNRC6B can modify cell proliferation and unusual fashion of cell adhesion in hepatoma cell lines

**Figure 4:**
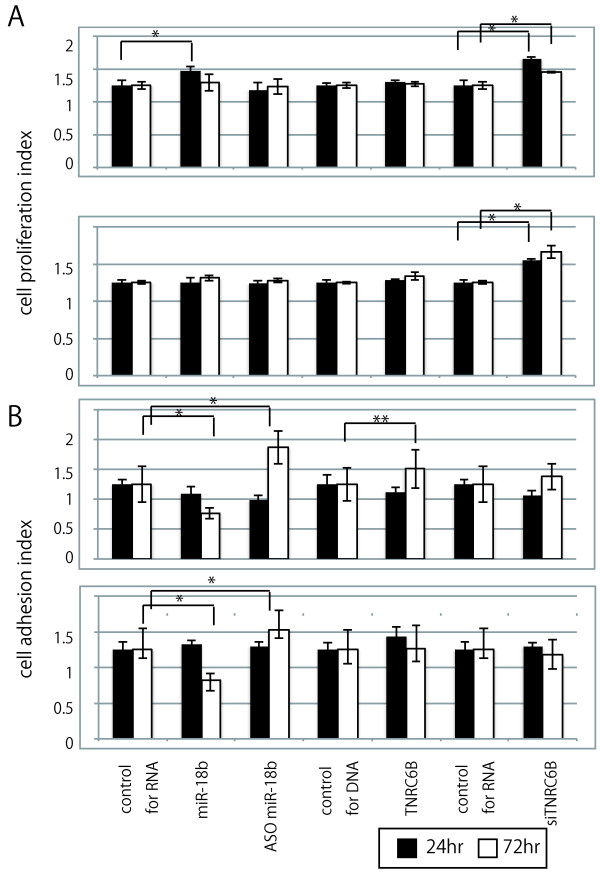
**The association between miR-18b and TNRC6B expression pattern and the cell proliferation and adhesion in hepatoma cell lines. A**) Cell proliferation index in Huh7 and Li7 cells respectively, after over-expression of miR-18b or TNRC6B, or suppression of miR-18b or TNRC6B for 24 or 72 hr. The data shown are means ± SD of three independent experiments. **B**) Cell adhesion index in Huh7 and Li7 cells respectively after over-expression of miR-18b or TNRC6B, or suppression of miR-18b or TNRC6B for 24 or 72 hr. The data shown are means ± SD of three independent experiments. Asterisk and double asterisk indicate a statistically significant difference of (p < 0.05) and (p < 0.01), respectively.

Over-expression of miR-18b and inhibition of TNRC6B by siRNA in human hepatoma cell lines Huh7, showed the progression of cell proliferation. Inhibition of TNRC6B by siRNA in both Huh7 and Li7, showed the progression of cell proliferation (p < 0.05) (Figure 4A). In both cell lines, over-expression or inhibition of miR-18b showed deceleration or acceleration of cell adhesion respectively (p < 0.05). Over-expression of TNRC6B by siRNA showed acceleration of cell adhesion in Huh7 (p < 0.01); however, over–expression of TNRC6B by siRNA also showed a similar acceleration of cell adhesion in Li7 (Figure 4B). These results indicated that over-expression of miR-18b and inhibition of TNRC6B, have the advantage of accelerating cell proliferation and decelerating cell adhesion.

### Over-expression of miR-18b in HCC is associated with poor prognosis

**Figure 5:**
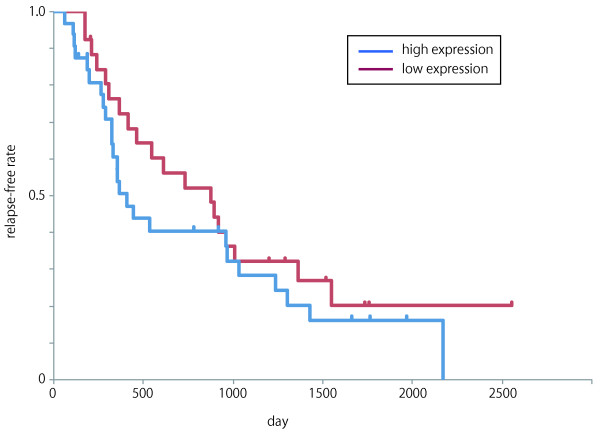
**miR-18b expression and relapse-free rate after surgical resection in 73HCC.** Kaplan–Meier curves showing the percentage of relapse-free HCC patients after surgical resection grouped on the basis of their median miR-18b expression level.

We then analyzed the prognosis of 73 HCC whose progress was monitored after surgery resection. Kaplan-Meier survival analysis and log-rank test demonstrated a significant difference in the outcomes of patients who were divided into two groups based on their median miR-18b expression level (p < 0.05). Specifically, patients with high expression of miR-18b had significantly lower survival rate than patients with low miR-18b expression. While miR-18b expression was associated with the relapse-free rate after surgical resection, we found that it did not significantly affect the overall survival rate (Figure 5 and Additional file 1: Table S2).

### Comparison between clinical background and miRNA expression pattern

To ascertain if any connection exists between miRNA expression and clinical background, we compared miRNA expression with tumor size, gender, age and background of HCC. Since a 20 mm diameter tumor is standard for liver cancer in the early stage, we compared the miRNA expression for HCC larger than 20 mm with those smaller than 20 mm. Three miRNAs were extracted based on two criteria: fold change 0.5 > or 2.0<, and *t*-test p < 0.05. The expression level of miR-1471 in small HCC was significantly higher than in large HCC, and the expression level of miR-499-5p and miR-609 in small HCC was significantly lower than in large HCC (Table 3).

**Table 3:** The relationship between several clinical factors and expression pattern of miRNAs

		
**tumor size**	20mm>/20mm<	
	fold change	p-value
hsa-miR-499-5p	0.35	0.01313
hsa-miR-1471	2.67	0.01665
hsa-miR-609	0.42	0.02072
**gender**	female/male	
	fold change	p-value
hsa-miR-765	0.34	0.01788
hsa-miR-622	0.31	0.02970
hsa-miR-1300	0.48	0.03314
**background of HCC**	CH/LC	
	fold change	p-value
hsa-miR-181d	2.48	0.00192
hsa-miR-542-3p	2.30	0.00945
hsa-miR-519e	2.08	0.01064
hsa-miR-936	0.17	0.01736
**age**	66years old > 67y.o.	
	fold change	p-value
hsa-miR-654-5p	3.88	0.00014
hsa-miR-493*	3.10	0.00016
hsa-miR-410	2.93	0.00029
hsa-miR-376a*	2.66	0.00072
hsa-miR-758	2.87	0.00073
hsa-miR-381	2.39	0.00094
hsa-miR-543	2.07	0.00119
hsa-miR-539	3.06	0.00124
hsa-miR-487b	2.02	0.00186
hsa-miR-337-5p	2.54	0.00195
hsa-miR-136*	2.79	0.00246
hsa-miR-154*	2.27	0.00337
hsa-miR-330-3p	2.44	0.00759
hsa-miR-421	2.45	0.01282

We also identified miRNAs with expression levels that varied according to gender and age. Namely, miR-765, miR-622, and miR-1300 had significantly lower expression levels in female HCC than in male HCC (Table 2). In regards to age, we discovered that the expression levels of 14 miRNAs (miR-654-5p, miR-493*, miR-410, miR-376a*, miR-758, miR-381, miR-543, miR-539, miR-487b, miR-337-5p, miR-136*, miR-154*, miR-330-3p, and miR-421) were significantly higher in HCC up to 66 years old than in HCC over 67 years old. The average age of the HCC subjects was 66.8 years old (Table 3).

Finally, when miRNA expression pattern was linked to the cause of HCC, we found that the expression level of miR-181d, miR-542-3p, and miR-519e in HCC derived from CH was significantly higher than in HCC from liver cirrhosis (LC). Additionally, the expression level of miR-939 in HCC derived from CH was significantly lower than in HCC from LC (Table 3). However, we found no significant correlation between the expression pattern of miR-18b and tumor size, age, gender, and background of HCC.

## Discussion

In the present study we established that in HCC miRNA was differentially expressed according to the grade of histological differentiation, recurrence of HCC after resection, tumor size, HCC background, age, and gender. In addition, we also established that over-expression of miR-18b and down-regulation of TNRC6B was closely associated with the proliferation of HCC. Extending our analysis to all miRNAs made it clear that the expression level of several miRNAs correlated with the progress of HCC. Recent reports have asserted that when distinguishing several diseases using miRNA profiling in the blood, diagnostic accuracy is higher when relative large numbers of miRNAs is used [19]. Therefore, performing a comprehensive miRNA analysis can be a shortcut for investigating novel biomarker for HCC.

Previously, we built a miRNA microarray based on the miRbase ver. 5.0 and reported that miR-92, miR-20, miR-18 and precursor miR-18 had significantly high expression in poorly differentiated HCC samples, moderate expression in moderately differentiated HCC and low expression in well-differentiated HCC. In contrast, miR-99a expression exhibited a positive correlation with the degree of tumor differentiation [9]. In the present study, we used a miRNA microarray referenced on the miRbase ver. 14.0 and showed that the expression of miR-221, miR-18a, miR-18b, and miR-423-5p in poorly differentiated HCC were significantly higher than in well differentiated HCC, and 8 miRNAs (miR-455-3p, miR-1914*, miR-100, miR-215, miR-122*, let-7b, miR-22 and miR-99a) in poorly differentiated HCC were expressed significantly lower than in well differentiated HCC. The expression pattern of miR-18, 22, 99, 221 in HCC observed in this study are similar to that noted in our previous reports [9].

Considering our miRNA profiling in HCC based on a variety of clinical information (grade of histological differentiation, recurrence of HCC after resection, size of tumor, the background of liver disease, age, and gender) and our analysis of the efficiencies of miRNAs in relation to cancer cell proliferation or adhesion, we strongly believe that miR-18b may be the gene with the most potential as a biomarker for diagnosing, prognosing or elucidating molecular pathogenesis.

Other studies have indicated that over-expression of both miR-221 and miR-18a is associated with hepatocarcinogenesis [20,21] and that over-expression of miR-221 is related to the advancement of tumor stages and metastasis [22]. Down-regulation of miR-22 has proliferative effect on HCC [23]. Prior studies using borderline tissue from colorectal liver metastases have validated the liver invasion front-specific down-regulation of miR-19b, miR-194, let-7b and miR-1275, and the tumor invasion front-specific down-regulation of miR-143, miR-145, let-7b and miR-638 [24].

Perturbations of miRNA networks are linked to a wide variety of pathological processes, including cardiovascular diseases and cancer. In this study we showed that a) over-expression of miR-18b was associated with poor prognosis of HCC; b) miR-18b has the ability to control the expression of TNRC6B gene as a target; and c) over-expression of miR-18b and down-regulation of TNRC6B showed malignant potential for hepatocarcinogenesis.

TNRC6B, a RNA recognition motif–containing protein, is localized to mRNA-degrading cytoplasmic P bodies and is functionally required to mediate miRNA-guided mRNA cleavage [25]. TNRC6B is expressed in many normal tissues including the prostate and is more suppressed in hormone-refractory metastatic prostate cancer than in prostate carcinoma [26]. Polymorphism of the promoter region of TNRC6B was also associated with prostate cancer [27]. Alterations in TNRC6B gene expression due to genetic variations might perturb the levels of mRNA species normally under its control and therefore contribute to carcinogenesis. Therefore, aberrant expression of TNRC6B might also contribute to hepatocarcinogenesis. This suggests that when miR-18b and TNRC6B are aberrantly expressed it is easy for oncogenesis to occur. Since our study revealed that TNRC6B did not correlate with recurrence, or survival rate of HCC, we speculate that TNRC6B may be regulated by a gene other than miR-18b. Detailed analysis is required in order to reach a conclusive decision.

## Conclusions

In this paper we presented the results of our miRNA expression profiling in HCC and highlighted the clinical and functional implications of miR-18b expression. Since down regulation of miR-18b and/or over-expression of TNRC6B inhibited cell proliferation and promoted cell adhesion, we propose miR-18b as a new diagnostic and prognostic miRNA marker for HCC progression. Our study provides a rationale for the classification and development of novel therapy for human HCC using miRNA profiling.

## Abbreviations

HCC: Hepatocellular carcinoma; TNRC6B: Trinucleotide repeat containing 6B; CHC: Chronic hepatitis C; LC: Liver cirrhosis; LNA: Locked nucleic acid; ASO: Antisense oligonucleotide

## Competing interests

The authors declare that they have no competing interests.

## Authors’ contributions

YM and AT conceived and designed the experiment; YM, HT, TK, SI, WW, NB, YK, and MK performed the experiment; TT and MT performed statistical analysis; YM, NK, TM, AM, NB, SK, and MK contributed to writing and editing the manuscript. All authors read and approved the manuscript.

## Pre-publication history

The pre-publication history for this paper can be accessed here:

http://www.biomedcentral.com/1471-2407/13/99/prepub

## Supplementary Material

Additional file 1: Table S1 Clinical background of HCC in detail. **Table S2**. Information of the surgical treatment and prognosis. **Table S3**. Inserter sequence of the miR-18b binding sequence of the TNRC6B 3′-UTR for reporter vector. Click here for file
