# Supplementary material for: The expression level of miR-18b in hepatocellular carcinoma is associated with the grade of malignancy and prognosis
Source: BMC Cancer. 2013 Mar 4;13:99. doi: 10.1186/1471-2407-13-99 (PMC3600030; doi:10.1186/1471-2407-13-99)
Supplement: Additional file 1: Table S1 — Clinical background of HCC in detail. Table S2. Information of the surgical treatment and prognosis. Table S3. Inserter sequence of the miR-18b binding sequence of the TNRC6B 3′-UTR for reporter vector. [file 1471-2407-13-99-S1.doc]

Table S1. Clinical background of HCC in detail

| code No. | histological differentiation | gender | age | HBsAg | anti-  HCV | tumor size (mm) | surround histology  of HCC | AFP | PIVKA-II |
| --- | --- | --- | --- | --- | --- | --- | --- | --- | --- |
| 1 | well | M | 67 | - | + | 18 x 15 | LC | 121.1 | 0.06 |
| 4 | well | M | 62 | - | + | 61 x 51 | CH | 7.9 | 0.52 |
| 11 | well | M | 56 | - | + | 12 x 10 | LC | 206 | <0.06 |
| 22 | well | M | 66 | - | + | 25 x 25 | CH | 10.1 | 0.29 |
| 64 | well | M | 66 | - | + | 14ｘ13 | LC | 321 | <0.06 |
| 70 | well | M | 72 | - | + | 20 x 25 | LC | 45.7 | NI |
| 108 | well | M | 69 | - | + | 18x18 | LC | 429.7 | 13 |
| 143 | poorly | M | 74 | - | + | 28x26 | CH | 58.6 | 228 |
| 144 | poorly | M | 69 | - | + | 21x20 | LC | 10.8 | 411 |
| 149 | poorly | M | 61 | - | + | 21x19 | LC | 484 | 30 |
| 150 | well | M | 52 | - | + | 28x25 | LC | 124 | 16 |
| 151 | poorly | M | 68 | - | + | 22x21 | LC | 269 | 110 |
| 178 | moderately | M | 67 | - | + | 42x 32 | LC | 20.4 | 203 |
| 183 | poorly | M | 56 | - | + | 50 x 35 | LC | 6.8 | 50 |
| 188 | well | M | 66 | - | + | 18 x 19 | CH | 21 | 24 |
| 190 | poorly | F | 76 | - | + | 28 x 21 | LC | 12.6 | 638 |
| 198 | poorly | M | 60 | - | + | 40 x 30 | CH | 27.7 | 1595 |
| 200 | poorly | M | 73 | - | + | 19x18 | CH | 123 | 122 |
| 202 | moderately | M | 76 | - | + | 100x90 | CH | 3263.5 | 20000< |
| 208 | poorly | M | 76 | - | + | 58 x 44 | CH | 2.5 | 945 |
| 211 | well | M | 79 | - | + | 24 x 30 | LC | 7.8 | 24 |
| 216 | well | M | 69 | - | + | 17 x 15 | CH | 7.3 | 19 |
| 219 | moderately | M | 68 | - | + | 20 x 18 | CH | 3.4 | 232 |
| 221 | moderately | M | 66 | - | + | 16 x 18 | CH | 3.2 | 41 |
| 222 | poorly | F | 68 | - | + | 67 x 52 | LC | 14.2 | 20000 |
| 223 | moderately | M | 76 | - | + | 25 x 20 | CH | 7.6 | 70 |
| 224 | poorly | M | 69 | - | + | 48 x 30 | LC | 3.4 | 9 |
| 228 | moderately | M | 76 | - | + | 22 x 15 | LC | 57.7 | 56 |
| 229 | poorly | M | 74 | - | + | 15 x 13 | LC | 207.5 | 29 |
| 235 | poorly | M | 70 | - | + | 20 x 22 | LC | 4.6 | 87 |
| 238 | moderately | M | 67 | - | + | 15 x 14 | LC | 83 | 14 |
| 241 | moderately | F | 85 | - | + | 50×42 | LC | 50 | 581 |
| 246 | moderately | M | 68 | - | + | 37×37 | LC | 3.1 | 28 |
| 248 | moderately | M | 73 | - | + | 18×15 | CH | 15 | 11 |
| 249 | moderately | F | 72 | - | + | 40×44 | LC | 36 | 170 |
| 251 | moderately | M | 68 | - | + | 13×13 | LC | 5.9 | 13 |
| 253 | moderately | M | 68 | - | + | 20 x 20 | LC | 25 | 15 |
| 258 | moderately | M | 59 | - | + | 20×18 | CH | 11 | 39 |
| 260 | moderately | M | 62 | - | + | 20 x 20 | CH | 5.1 | 5 |
| 261 | poorly | M | 69 | - | + | 47×43 | CH | 18.9 | 3850 |
| 268 | poorly | M | 66 | - | + | 42×26 | CH | 2062 | 106 |
| 269 | poorly | M | 71 | - | + | 80×30 | CH | 2.7 | 217 |
| 271 | moderately | M | 55 | - | + | 65×40 | CH | 11.4 | 1076 |
| 272 | moderately | F | 72 | - | + | 24×21 | CH | 3.8 | 10 |
| 274 | poorly | M | 67 | - | + | 54×45 | LC | 176.6 | NI |
| 275 | well | M | 62 | - | + | 10×9 | LC | 11 | 79 |
| 277 | moderately | M | 60 | - | + | 45×45 | LC | 2.2 | 1909 |
| 278 | poorly | M | 71 | - | + | 14×8 | CH | 49.4 | 13 |
| 281 | poorly | M | 69 | - | + | 16×20 | CH | 2 | 17 |
| 282 | poorly | M | 62 | - | + | 20×20 | LC | 1124 | NI |
| 295 | poorly | M | 64 | - | + | 40×36 | CH | 3 | 36 |
| 297 | moderately | F | 63 | - | + | 30×25 | CH | 1774 | 23 |
| 299 | moderately | M | 69 | - | + | 22×17 | LC | 23.6 | 251 |
| 310 | moderately | M | 71 | - | + | 42×40 | CH | 5.2 | 202 |
| 314 | moderately | F | 67 | - | + | 25×28 | CH | 811.6 | 233 |
| 315 | moderately | M | 69 | - | + | 30×16 | LC | 200 | NI |
| 318 | moderately | M | 73 | - | + | 38×34 | CH | NI | 64 |
| 319 | moderately | M | 70 | - | + | 42×35 | LC | 48 | 29 |
| 321 | moderately | M | 72 | - | + | 47×30 | CH | 4.2 | 307 |
| 322 | moderately | M | 70 | - | + | 79×70 | LC | 5.5 | 7158 |
| 324 | poorly | M | 65 | - | + | 28×20 | CH | 45.9 | 35 |
| 325 | poorly | M | 63 | - | + | 30×20 | CH | 650 | 153 |
| 327 | poorly | M | 66 | - | + | 18×15 | LC | 106.6 | NI |
| 328 | poorly | M | 72 | - | + | 12×10 | LC | 145.5 | 42 |
| 329 | moderately | Ｍ | 68 | - | + | 39×44 | LC | 4 | 281 |
| 330 | poorly | Ｆ | 73 | - | + | 18×18 | LC | 254 | NI |
| 333 | moderately | Ｍ | 72 | - | + | 24×18 | LC | 9.1 | 77 |
| 335 | moderately | Ｍ | 65 | - | + | 30×25 | LC | 10.6 | 1174 |
| 336 | moderately | Ｍ | 76 | - | + | 35×32 | NI | 15.9 | 570 |
| 340 | poorly | Ｍ | 73 | - | + | 15×14 | CH | 486.8 | 71 |
| 345 | poorly | Ｍ | 57 | - | + | 55×55 | LC | 1700.7 | 1646 |
| 346 | moderately | Ｆ | 74 | - | + | 27×20 | NI | 12.8 | 204 |
| 347 | poorly | Ｍ | 73 | - | + | 35×27 | CH | 4 | 162 |
| CU-070 | moderately | M | 53 | - | + | 60x55 | LC | 3510 | NI |
| CU-083 | moderately | M | 49 | + | - | 80x72 | LC | 143830 | 0.9 |
| CU-085 | moderately | M | 55 | - | + | 22x20 | LC | 113 | 0.06 |
| CU-087 | well | M | 62 | - | + | 120x80 | NI | NI | NI |
| CU-089 | moderately | F | 61 | - | + | 40x33 | CH | 400 | 0.06 |
| CU-091 | moderately | M | 64 | - | + | 27x25 | CH | 5 | 0.06 |
| K-023 | moderately | M | 47 | + | - | NI | LC | 3500 | 2.2 |
| K-122 | well | F | 69 | + | - | NI | LC | 1.1 | 0.243 |
| K-160 | moderately | M | 54 | - | + | NI | NI | NI | NI |
| K-177 | moderately | M | 68 | - | + | NI | NI | 13 | 29 |
| K-183 | well | M | 73 | - | + | NI | LC | 6.3 | 46 |
| K-204 | poorly | M | 56 | - | + | NI | LC | 3700 | 0.097 |
| K-209 | moderately | M | NI | NI | NI | NI | NI | NI | NI |
| O-001 | well | M | 73 | - | + | NI | CH | 11.5 | 33 |
| O-002 | moderately | M | 63 | - | + | NI | LC | 20.8 | 3423 |
| O-003 | well | F | 59 | + | - | NI | CH | 5.2 | 11 |
| O-004 | moderately | F | 76 | - | + | NI | CH | 49 | 154 |
| O-005 | moderately | M | 69 | - | + | NI | LC | 168.4 | 15 |
| O-006 | moderately | M | 55 | - | + | NI | CH | 10.2 | 408 |
| O-086 | moderately | F | 64 | - | + | NI | LC | 218 | 32 |
| O-088 | moderately | M | 74 | + | - | NI | LC | 13.4 | 7991 |
| O-089 | moderately | M | 68 | - | + | NI | LC | 8 | 25 |
| O-090 | moderately | M | 70 | - | + | NI | LC | 686.3 | 962 |
| O-092 | moderately | M | 56 | + | - | NI | CH | 2.7 | 790 |
| O-093 | well | F | 72 | - | + | NI | LC | 51.5 | 39 |
| O-098 | moderately | M | 77 | - | + | NI | LC | 164.5 | 64815 |
| O-100 | moderately | M | 65 | + | - | NI | CH | 2.7 | 18 |
| O-103 | moderately | M | 69 | - | - | NI | CH | 5241 | 4139 |
| O-104 | moderately | M | 59 | + | - | NI | CH | 179.1 | 329 |
| O-105 | moderately | M | 79 | - | - | NI | CH | 16150 | 6334 |
| O-108 | well | F | 67 | - | + | NI | CH | 7.8 | 46 |
| O-109 | well | F | 69 | - | + | NI | LC | 95.5 | 18 |
| O-110 | moderately | M | 36 | + | - | NI | LC | 287.5 | 54 |
| O-111 | moderately | M | 69 | - | - | NI | LC | 26.1 | 31 |
| O-112 | moderately | M | 59 | - | + | NI | LC | 5.6 | 24 |
| O-113 | moderately | M | 71 | - | + | NI | CH | 14.4 | 23 |
| O-115 | moderately | M | 76 | - | + | NI | CH | 6.4 | 560 |

Abbreviation NI; no information, CH; chronic hepatitis, LC; liver cirrhosis, tumor size; major axis x minor axis

Table S2. Information of the surgical treatment and prognosis

| code No. | date of operation | confirmed life-and-death date | survival period (day) | date of recurrence | unrecurred period (day) |
| --- | --- | --- | --- | --- | --- |
| 1 | 1997.5.19 | 2002.10.2 | 1962 | 1998.5.21 | 367 |
| 4 | 1995.4.24 | 1995.11.11 | 201 | 1995.11.11 | 201 |
| 11 | 1993.8.30 | 1998.1.2 | 1586 | 1994.2.17 | 171 |
| 22 | 1996.3.4 | 1997.11.2 | 608 | 1996.6.18 | 106 |
| 64 | 1997.6.25 | 2010.5.31 | 4723 | 1997.10.21 | 118 |
| 70 | 1997.9.1 | 2006.2.12 | 3086 | 2000.2.10 | 892 |
| 108 | 1998.7.13 | 2007.5.17 | 3230 | 2001.1.18 | 920 |
| 143 | 1999.4.26 | 2001.6.15 | 781 | 2000.2.10 | 290 |
| 144 | 1999.6.16 | 2009.4.26 | 3602 | 2003.1.10 | 1304 |
| 149 | 1999.4.12 | 2005.5.15 | 2225 | 2000.6.27 | 442 |
| 150 | 1998.11.9 | 2002.10.20 | 1441 | 2000.4.25 | 533 |
| 151 | 1999.9.22 | 2001.10.26 | 765 | NI | NI |
| 178 | 2001.8.20 | 2010.5.31 | 3206 | 2005.11.16 | 1549 |
| 183 | 2001.7.23 | 2008.7.14 | 2548 | 2004.3.10 | 961 |
| 188 | 2002.9.30 | 2010.5.31 | 2800 | 2003.8.19 | 323 |
| 190 | 2002.9.30 | 2004.10.23 | 754 | 2003.9.16 | 351 |
| 198 | 2003.6.4 | 2010.5.31 | 2553 | 2010.5.31 | 2553 |
| 200 | 2002.12.2 | 2010.5.31 | 2737 | NI | NI |
| 202 | 2003.8.18 | 2003.11.15 | 89 | NI | NI |
| 208 | 2003.11.5 | 2010.5.31 | 2399 | 2007.3.22 | 1233 |
| 211 | 2003.12.8 | 2004.6.11 | 186 | 2004.6.11 | 186 |
| 216 | 2004.1.7 | 2010.5.31 | 2336 | NI | NI |
| 219 | 2004.2.18 | 2004.12.15 | 301 | 2004.11.5 | 261 |
| 221 | 2003.5.29 | 2010.5.31 | 2558 | 2010.5.31 | 2558 |
| 222 | 2003.6.11 | 2004.1.6 | 209 | 2003.10.3 | 114 |
| 223 | 2003.6.23 | 2006.12.31 | 1287 | 2006.12.31 | 1287 |
| 224 | 2003.6.30 | 2010.5.31 | 2527 | 2007.3.22 | 1361 |
| 228 | 2004.5.26 | NI | NI | NI | NI |
| 229 | 2004.6.2 | 2008.7.5 | 1494 | 2005.3.18 | 289 |
| 235 | 2004.6.21 | 2010.5.31 | 2170 | 2010.5.31 | 2170 |
| 238 | 2004.8.11 | 2010.5.31 | 2119 | 2005.6.15 | 308 |
| 241 | 2004.10.13 | 2005.3.1 | 139 | 2005.3.1 | 139 |
| 246 | 2004.11.22 | 2010.5.31 | 2016 | 2007.8.24 | 1005 |
| 248 | 2005.1.12 | 2010.5.31 | 1965 | 2010.5.31 | 1965 |
| 249 | 2005.1.17 | 2010.5.31 | 1960 | 2008.12.15 | 1428 |
| 251 | 2005.1.31 | 2010.5.31 | 1946 | NI | NI |
| 253 | 2005.3.2 | 2010.5.31 | 1916 | 2007.7.23 | 873 |
| 258 | 2005.4.13 | 2010.5.31 | 1874 | NI | NI |
| 260 | 2005.5.31 | 2010.5.31 | 1826 | NI | NI |
| 261 | 2005.5.16 | 2006.2.24 | 284 | 2005.7.12 | 57 |
| 268 | 2005.8.3 | 2010.5.31 | 1762 | 2010.5.31 | 1762 |
| 269 | 2005.9.2 | 2010.5.31 | 1732 | 2010.5.31 | 1732 |
| 271 | 2005.10.19 | 2008.6.19 | 974 | 2007.4.16 | 544 |
| 272 | 2005.10.26 | NI | NI | NI | NI |
| 274 | 2005.8.1 | 2007.9.24 | 784 | 2006.2.5 | 188 |
| 275 | 2005.8.8 | 2010.5.11 | 1737 | 2010.5.31 | 1757 |
| 277 | 2005.9.7 | 2007.11.13 | 797 | NI | NI |
| 278 | 2005.10.19 | 2010.5.31 | 1685 | 2008.6.2 | 957 |
| 281 | 2005.11.14 | 2010.5.31 | 1659 | 2010.5.31 | 1659 |
| 282 | 2005.11.21 | 2010.5.31 | 1652 | NI | NI |
| 295 | 2006.2.15 | 2010.5.31 | 1566 | 2007.2.15 | 365 |
| 297 | 2006.4.3 | 2010.5.31 | 1519 | 2010.5.31 | 1519 |
| 299 | 2006.4.24 | 2010.5.31 | 1498 | 2008.12.15 | 966 |
| 310 | 2006.7.19 | NI | NI | NI | NI |
| 314 | 2006.5.22 | 2008.1.21 | 609 | 2008.1.21 | 609 |
| 315 | 2006.6.19 | 2008.5.21 | 702 | 2007.6.7 | 353 |
| 318 | 2006.9.20 | 2010.5.31 | 1349 | 2007.10.30 | 405 |
| 319 | 2006.11.1 | 2008.10.17 | 716 | 2007.4.26 | 176 |
| 321 | 2006.12.20 | 2010.5.31 | 1258 | 2007.11.9 | 324 |
| 322 | 2007.1.15 | 2009.3.11 | 786 | 2008.3.4 | 414 |
| 324 | 2007.1.24 | 2007.11.13 | 293 | 2007.8.24 | 212 |
| 325 | 2007.5.30 | 2008.12.17 | 567 | 2007.12.13 | 197 |
| 327 | 2007.6.25 | 2010.5.31 | 1071 | 2008.5.19 | 329 |
| 328 | 2007.6.27 | 2010.5.31 | 1069 | 2009.6.27 | 731 |
| 329 | 2007.2.19 | 2010.5.31 | 1197 | 2010.5.31 | 1197 |
| 330 | 2007.2.20 | 2009.2.10 | 721 | NI | NI |
| 333 | 2007.3.28 | 2010.5.31 | 1160 | 2010.1.21 | 1030 |
| 335 | 2007.4.9 | 2010.5.31 | 1148 | 2008.7.14 | 462 |
| 336 | 2007.10.31 | 2010.5.31 | 943 | 2008.6.27 | 240 |
| 340 | 2007.11.26 | 2010.5.31 | 917 | 2010.5.31 | 917 |
| 345 | 2007.6.25 | 2009.5.21 | 696 | 2008.3.25 | 274 |
| 346 | 2007.7.9 | 2010.5.31 | 1057 | NI | NI |
| 347 | 2008.4.9 | 2010.5.31 | 782 | 2010.5.31 | 782 |

Abbreviation NI; no information

Table S3. Inserter sequence of the miR-18b binding sequence of the TNRC6B 3’-UTR for reporter vector

TNRC6B-WS

5’-CTAGTgcaggtataacttagcgaagacttttaagtatt**gcacctt**tttttgatttttgA-3’

TNRC6B-WAS

5’-AGCTTcaaaaatcaaaaa**aaggtgc**aatacttaaaagtcttcgctaagttatacctgcA-3’

TNRC6B-MS

5’-CTAGTgcaggtataacttagcgaagacttttaagtattgcaggaatttttgatttttgA-3’

TNRC6B-MAS

5’-AGCTTcaaaaatcaaaaattcctgcaatacttaaaagtcttcgctaagttatacctgcA-3’

Capital letter is indicated that 5’ of sense is Spe I recognition site and 3’ of sense is HindIII recognition site. Bold letter indicates miR-18b binding site. Underlined letter is mutated miR-18b binding site.
